# Development of a dynamic pulsatile phantom for the photoplethysmographic waveform at the radial artery

**DOI:** 10.1117/1.JBO.30.11.117001

**Published:** 2025-11-07

**Authors:** Tananant Boonya-ananta, Andres J. Rodriguez, Ajmal Ajmal, Amanda N. Sanchez, JunZhu Pei, Ernesto Rodriguez, Abiel Vasallo Veliz, Christian Suastegui, Nicole Paz, Jessica C. Ramella-Roman

**Affiliations:** a Florida International University, Department of Biomedical Engineering, Miami, Florida, United States; b Florida International University, Department of Mechanical Engineering, Miami, Florida, United States; c Florida State University, Department of Chemical and Biomedical Engineering, Tallahassee, Florida, United States; d University of Miami, Department of Mechanical and Aerospace Engineering, Coral Gables, Florida, United States; e Florida International University, Herbert Wertheim College of Medicine, Miami, Florida, United States

**Keywords:** dynamic phantom, photoplethysmography, wearables

## Abstract

**Significance:**

Cardiovascular disease remains one of the leading causes of death in the United States. Wearable optical systems are known to have errors and biases for individuals with different skin tones as well as different levels of obesity. By enabling the development and validation of wearable technologies across diverse populations, we advance equitable healthcare solutions and foster the creation of more reliable, personalized health monitoring systems.

**Aim:**

We aim to develop a dynamic wrist phantom replicating the radial artery pulse, addressing physiological variations such as skin tone and obesity that impact wearable health technologies.

**Approach:**

A silicone-based phantom mimics human tissues’ mechanical and optical properties. A cam-driven pulsatile flow system simulated physiological blood flow, with key waveform features controlled by mechanical components. Optical properties were adjusted using titanium dioxide and carbon black to match Fitzpatrick skin tones I to VI, whereas radial artery depth variations simulated the effects of obesity. The phantom system incorporated a blood-mimicking fluid to replicate the optical absorption characteristics of whole blood.

**Results:**

The phantom successfully replicated photoplethysmography (PPG) waveforms at heart rates ranging from 59 to 118 beats per minute, demonstrating physiologically representative features such as systolic and diastolic peaks. Signal degradation was observed with increasing vessel depth and darker skin tones, consistent with real-world challenges in wearable device accuracy. The alternating signal/baseline signal ratio of the PPG signal decreased by up to 77.8% for darker skin tones and deeper vessels. The phantom also validated its performance against commercial wearables, supporting its utility in device testing.

**Conclusions:**

This dynamic wrist phantom provides a robust platform for evaluating optical devices under controlled and representative conditions, addressing critical gaps in inclusivity and accuracy.

## 1 Introduction

Cardiovascular disease (CVD) is one of the current leading causes of death in the United States, as reported by the Centers for Disease Control and Prevention. This, in turn, costs the United States ∼$240 billion each year, including health care services, medication, treatment, and losses in productivity due to death. The underlying cause of CVD is interlinked with various conditions such as diabetes, obesity, and behavioral factors, including an unhealthy diet and/or physical inactivity, as well as excessive alcohol consumption and tobacco use.^1^ The most common cardiovascular diseases are coronary artery disease, heart attack, and stroke. Annually, this amounts to over 20% of all deaths in the United States.^1^ The underlying condition of chronic hypertension (high blood pressure) also significantly increases the risk factors for the development of cardiovascular disease.

Various technologies exist to investigate the cardiac circuit and to understand pressure and flow parameters, disease status, or therapeutic processes.^2^ Yet, more effective and personalized cardiovascular care is becoming prevalent with advancements in wearable and point-of-care devices,^3^ which often rely on noninvasive optical technology.

Unfortunately, recent work has shown how optical devices used in wearables for photoplethysmography (PPG) and pulse oximetry suffer from or have higher errors when utilized by individuals with elevated skin tone.^4^ Obesity is known to introduce anatomical and physiological variations at the wrist, including increased subcutaneous adipose tissue, changes in vascular structure, and altered skin hydration and thickness. These changes affect the optical path and can degrade the quality of PPG signals. Although heart rate estimation using PPG remains relatively robust, other derived metrics,^5^^,^^6^ such as blood oxygenation, caloric expenditure, and vascular compliance, are more susceptible to error. Previous work has reviewed obesity-associated anatomical changes and their predicted influence on optical signal acquisition.^7^ However, there remains a significant gap in the literature regarding direct measurements of obesity-induced changes in tissue optical properties and their quantitative impact on commercial wearable performance.

**Fig. 1:**
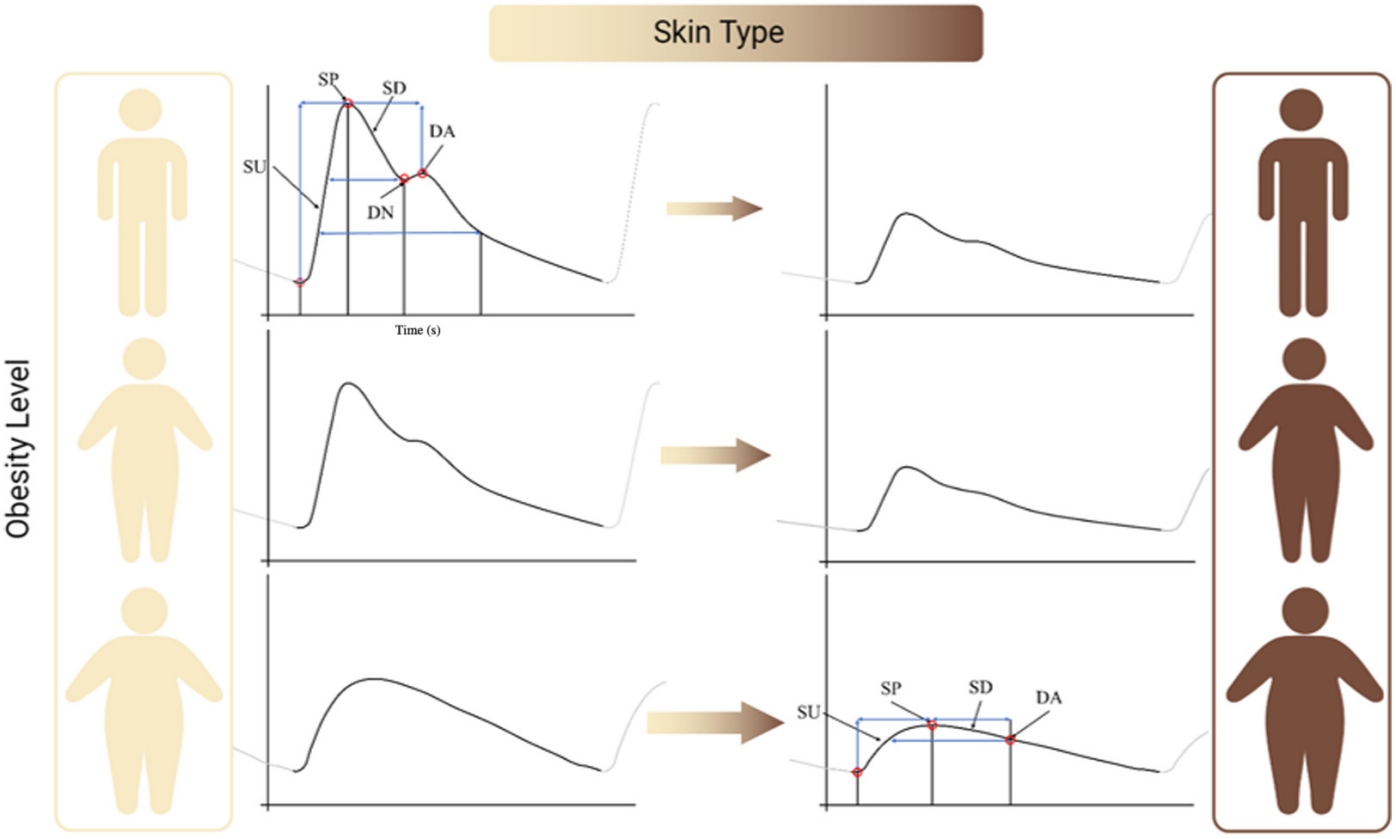
Variation of the PPG waveform from the effects of skin tone and obesity. SU, systolic upstroke; SP, systolic peak; SD, systolic decline; DN, dicrotic notch; and DA, diastolic peak.

Furthermore, we have shown through computational modeling that PPG^7^^–^^10^ can be influenced by factors such as age, BMI, sex, and skin color. Changes to the PPG waveform (Fig. 1) due to obesity and skin tone have been predicted to reduce overall signal quality.^8^^,^^9^^,^^11^ Nevertheless, these predictions are limited to skin tone and obesity and do not account for factors such as age and variations in vessel compliance.

We have shown a degradation of the PPG waveform due to both skin tone and obesity.^7^^–^^9^ However, capturing the full range of human variability in PPG signals, arising from differences in BMI, skin tone, age, and sex, can be challenging and time-consuming in real-world studies; therefore, a controlled environment for investigating these influences, such as computational modeling and phantom fabrication, may aid in validation and calibration.

Optical phantoms have been extensively used to test optical devices by mimicking geometry, biochemical and optical properties, and flow dynamics. The use of dynamic phantoms allows for replicating physiological characteristics and phenomena while controlling for variations that would otherwise exist among various individuals. Various authors have explored different techniques to mimic different anatomical parameters of the human body.

Skin optical phantoms have been extensively explored in literature, demonstrating different techniques of fabrication to match different properties, such as optical absorption and scattering coefficients, using a combination of absorbing and scattering agents^12^^–^^14^ combined with the inherent properties of the base material. Static optical phantoms have been developed in the past using gel-based, liquid, or solid structures, depending on their applications.^12^^,^^15^^–^^18^ Other skin-mimicking phantoms include multilayer phantoms^19^^,^^20^ representing a combination of the epidermis, dermis, and subcutaneous tissue with varying thickness and optical properties.^21^^–^^23^

Various authors^2^^,^^11^^,^^18^^,^^24^^–^^26^ have shown that silicone-based material, or polydimethylsiloxane, can be cast or 3D printed to provide controllable mechanical properties of biological tissue. Material selection for dynamic phantoms is critical to capture the relevant interaction between the biological behavior being replicated and the measurement method being employed. Crafting of the base skin/tissue phantom with an embedded vessel structure representing a major artery is commonly done through the combination of 3D modeling through computer-aided design software.

Dynamic phantoms used to replicate blood flow must represent the artery elasticity of the relevant area of measurement as well as the compliance of the surrounding tissue. In the case of a phantom for the optical system, the fluid must also match that of blood at the required wavelength,^27^ and the surrounding media must represent the surrounding biological tissue.

A dynamic phantom for flow simulation also consists of a mechanical/electrical device that controls the fluid system, modifying flow and/or pressure. Several commercially available flow systems can be obtained from manufacturers.^18^^,^^28^^,^^29^ These devices will have flow and pressure ranges and are only appropriate for specific applications. Some systems are designed specifically for carotid arterial flow and will not be physiologically representative of flow properties in other arteries, such as those found near anatomical sites where wearables are used. An alternative to these systems is a custom approach,^11^^,^^24^^,^^25^ which may be preferred due to specific requirements and costs.

Notably, Nomoni et al.^25^ investigate the relationship between source–detector separation and vessel depth on PPG signal simulating the brachial artery. This work has embedded a series of silicone tubes ranging from 3.4 to 24 mm in depth in a single homogenous gelatin-based medium. A pulsatile waveform is generated by a commercial system. Although this work demonstrated the impact of anatomical parameters on PPG signal quality, the phantom did not incorporate variations in optical properties related to skin tone or tissue composition. In addition, the use of a uniform gelatin medium does not reflect the layered structure or mechanical compliance of biological tissue. These areas present opportunities for expansion toward more physiologically and demographically representative phantom systems. This approach laid foundational work for the use of tissue-mimicking phantoms in evaluating wearable PPG systems and presents several opportunities for expansion, particularly in replicating physiologically realistic waveforms, simulating diverse demographic conditions, and enabling modular customization for system-level validation.

This research focuses on creating a dynamic, customizable optical phantom that accurately replicates PPG signal behavior at the radial artery, considering variations in skin tone and obesity. By incorporating these factors, the proposed phantom is a comprehensive tool to simulate the interaction among optical properties, vascular dynamics, and physiological variations. This system aims to enhance the development, testing, and validation of wearable health technologies, particularly for populations often underrepresented in clinical research. The work addresses a critical need for more inclusive and reliable health monitoring solutions, helping to bridge the gap between existing device limitations and the demand for equitable healthcare advancements.

## 2 Materials and Methods

**Fig. 2:**
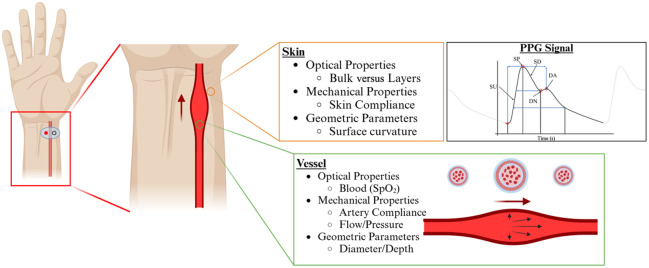
Design parameters for the dynamic radial artery phantom for PPG signal evaluation.

The development of the dynamic pulsatile phantom aims to replicate two critical parameters at the wrist: the mechanical behavior and the optical properties. The interaction of the optical signal requires the bulk material to be the same as the skin’s optical properties, as well as the working fluid having the absorption coefficient of oxygenated blood. The silicone tubing stiffness must fall within the same range as an actual radial artery blood vessel, and the surrounding silicone model should maintain similar compliance as standard bulk surrounding tissue. A diagram of design specifications is shown in Fig. 2.

### 2.1 Wrist Phantom

The critical control component is the characterization of the artery’s mechanical properties. The elasticity of the vessel largely determines the ability to accurately represent the radial artery’s performance under realistic physiological behavior. Mechanical properties of various major arteries^2^^,^^30^^–^^33^ (aorta, carotid, and coronary) have been studied in the literature, showing a wide range of stiffness moduli ranging from the 100’s of kilopascals to the 10’s of megapascals. Larger arteries (in the millimeter diameter range) tend to be less stiff than those with a smaller diameter. With the location at the wrist, the mechanical and geometrical properties of the radial artery are investigated^9^^,^^34^^–^^42^ (Table 1). It should be noted that the literature values presented in Table 1 are derived from studies that predominantly report measurements in adult populations without consistent stratification by race, skin tone, or BMI. As such, these values likely reflect general anatomical averages but may be biased toward lighter-skinned or nonobese individuals, unless otherwise specified, depending on the study cohort. This limitation underscores the importance of developing customizable phantoms like ours that can model a broader range of physiological variability, including those underrepresented in clinical research. Using these parameters, the phantom specifications will be outfitted to fall within the appropriate physiological ranges.

**Table 1:** Radial artery parameters in literature.

Property	Measurement	Values (units)	Article
Geometry	Diameter	Ø 2.71 ± 0.32 mm (left RA)	Madssen et al.^37^
	Diameter	Ø 3.3 ± 0.7 mm (external)	Nasr^38^
	Diameter	Male: Ø 2.68 ± 0.24 mm	Wahood et al.^42^
		Female: Ø 2.27 ± 0.27 mm	
Geometry/mechanical	Diameter	Ø 2.5 mm	Boonya-Ananta et al.^9^
	Depth	↧ 2.5 to 5.0 mm	
	Elastic modulus	E=0.75 MPa	
	Diameter	Ø 2.44 ± 0.36 mm	Girerd et al.^35^
	Wall thickness	t=0.27±.05 mm	
	Incremental modulus	$E_{\mathrm{inc}}=1$ to $4\times 10^{6}\;\text{dynes}/{\mathrm{cm}}^{2}$	
	Diameter	Ø 2.44 ± 0.17 to 2.59 ± 0.16 mm	Chamiot-Clerc et al.^34^
	Elastic modulus	E=2.2±0.6 to 9.9 ± 3.5 kPa	
	Diameter	Ø 2.53 ± 0.32 mm	Laurent et al.^36^
	Wall thickness	t=0.28±0.05 mm	
	Elastic modulus	E=2.68±1.81 to 3.28 ± 2.11 MPa	
Geometry/hemodynamics	Diameter/flow	Ø 2.87 ± 0.46 mm	Takayama et al.^40^
		$0.45\pm 0.29\;\mathrm{mL}/s/m^{2}$	
Hemodynamics	Flow	Q=0.9 to 15.3 mL/min	Trager et al.^41^

With these factors in mind, the first method of testing involved the selection of appropriate material for representing the radial artery. Different materials were selected based on their manufacturability to test for their mechanical properties. An EX5M manual test system performed a longitudinal displacement test on the custom silicone-based flexible material specimen. A standard ASTM D412^43^ is specially designed for the tensile testing of vulcanized rubber and thermoplastic elastomers and serves as a guideline for the characterization of this material’s properties.

**Fig. 3:**
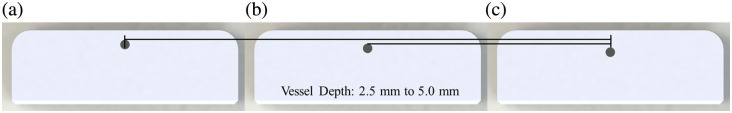
SolidWorks model of wrist phantom presentation of various arterial line depths from the tissue surface. Phantom is $60\times 60\times 20\;{\mathrm{mm}}^{3}$ in overall block size. (a) Vessel depth 2.5 mm, (b) intermediate vessel depth at 3.75 mm, and (c) deepest vessel depth 5.0 mm.

A silicone casting was used to model the wrist. First, several silicone castings were created with varying ratios of silicone base, and a curing agent (A + B) was created. The silicone samples were tested to determine the mixture that would yield mechanical properties similar to those of the bulk tissue area. Using SolidWorks, a 3D cast mold of a human wrist surface profile block was created for the phantom. A chosen silicone material property is used to model the radial artery within the silicone wrist during the casting process. The arterial vessel was created at a depth of 2.5 to 5.0 mm below the surface (Fig. 3).

To replicate the optical properties of the skin in the wrist phantoms, a silicone resin base is combined with titanium dioxide, ${\mathrm{TiO}}_{2}$, and carbon black (carbon, mesoporous nanopowder, Millipore Sigma)^12^^,^^13^ were added as the scattering and absorbing agents, respectively. Two initial stock solution mixtures are prepared for a desired skin tone by mixing carbon black and ${\mathrm{TiO}}_{2}$ as a percentage in weight of Resin Part B in two separate containers and diluting them to match the target optical properties pertaining to the Fitzpatrick skin tone groups. The mixture is poured into three circular 2.0 in. (51 mm) aluminum molds to achieve a 2.0 mm thick disc for optical properties measurement using a single integrating sphere and the inverse adding doubling method. At the same time, the mixture is poured into wrist phantom molds, each varying in radial artery vessel depths. The samples are then degassed in a vacuum chamber and cured overnight at room temperature. Another method to develop a stiffer range of mechanical properties for the wrist phantoms is done using a Form 3 SLA base 3D printer by adding a different mixture of carbon black and ${\mathrm{TiO}}_{2}$ to Formlabs Elastic 50A Resin V1^44^ and printed to have the same geometrical parameters as the mold-casted phantoms. Table 2 shows the optical properties range for bulk skin in three different Fitzpatrick skin tones^45^ desired at 660 nm.

**Table 2:** Fitzpatrick skin tone range at 660 nm.^45^

Fitzpatrick skin tone	Optical properties at 660 nm (${\mathrm{cm}}^{-1}$)	
	$\mu_{a}$	$\mu_{s}^{\prime }$
I to II	0.316	15.6
III to VI	0.446	22.1
V to IV	1.18	20.3

### 2.2 Flow and Pressure System Controls

**Fig. 4:**
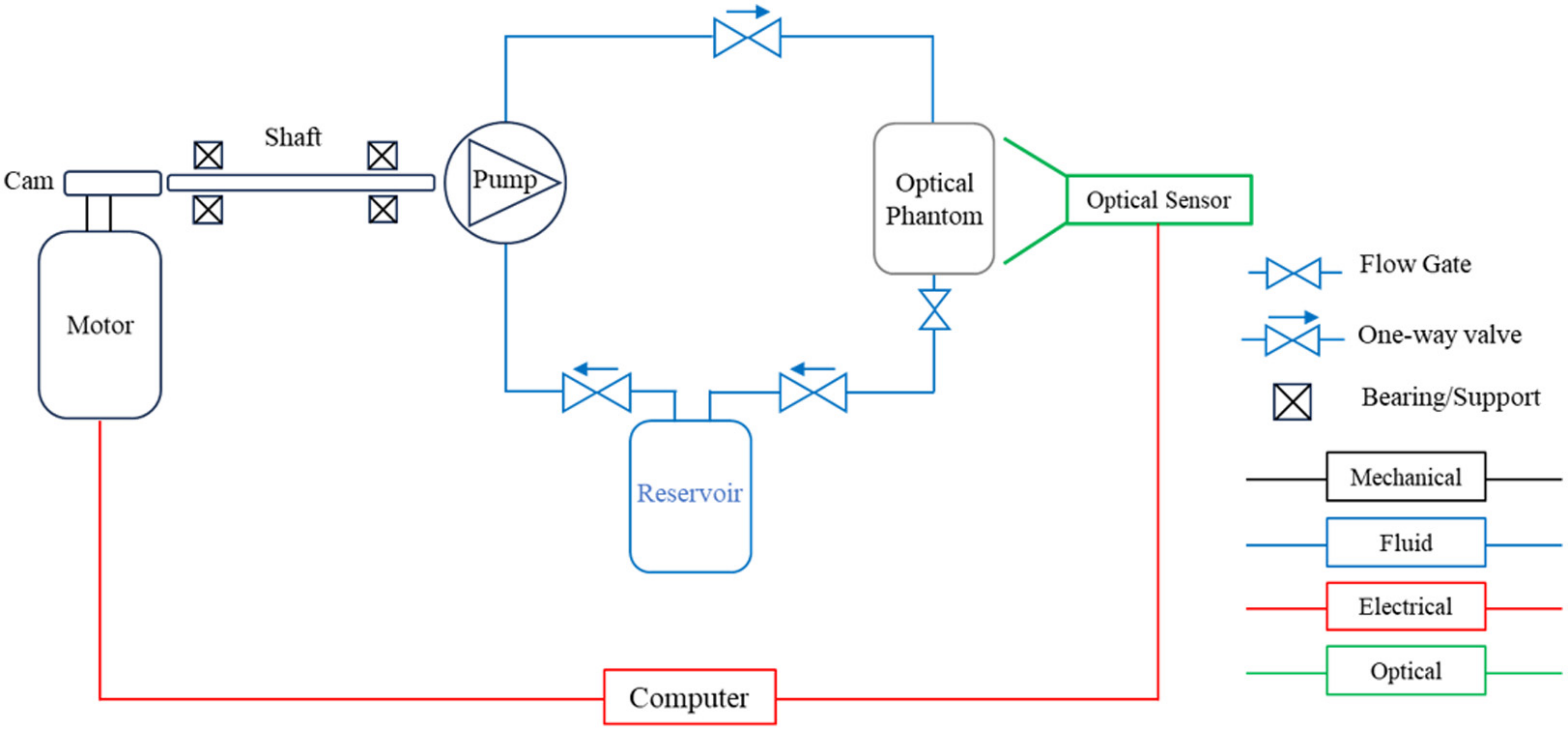
Phantom system flowchart; each colored line identifies a specific domain: mechanical, fluid, electrical, and optical. Each has its own controllable or measurable output and parameters.

The radial artery tubing was then integrated into the pulse flow system. Human pulse flow was simulated via a combination of mechanisms, as demonstrated in Fig. 4.

**Fig. 5:**
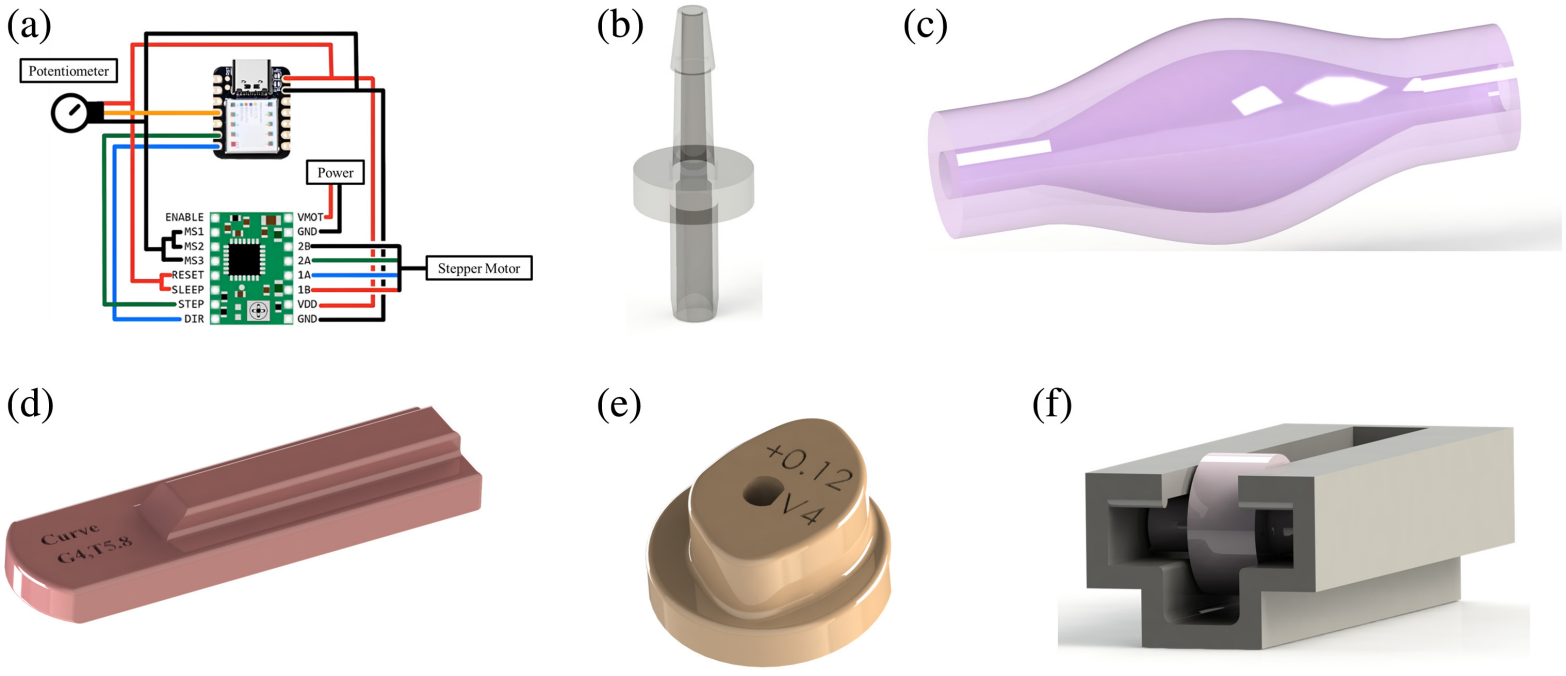
Control mechanism for the dynamic phantom. (a) Motor control circuit for 42-stepper motor and motor driver A4988, (b) custom Luer tip for the interface between system flow tubing and phantoms to optimize fitting and reduce flow cross-sectional area mismatch, (c) custom pump bulb to create pulsatile flow, (d) push shaft/cam follower to create compression of the bulb pump, (e) cam drive with custom profile to create arterial flow features, and (f) outlet pressurization clip for flow restriction after the optical wrist phantom.

Figure 4 shows the flowchart for the various paths that control the dynamic phantom system. The components in black outline the mechanical components, blue lines indicate the fluid flow system, red indicates the electrical signals, and green represents the collected optical systems. Figure 5 highlights critical components to generating, controlling, and shaping the pulse waveform captured at the optical phantom representative of the radial artery.

System pressure and flow are controlled via a peristaltic roller assembly powered by a Goso 42 Stepper Motor. The stepper motor has a resolution of 1.8 deg. The motor is controlled using an A4988 motor driver and an Arduino microcontroller board [Fig. 5(a)]. The stepper motor was powered by a 12 V, 2 A AC adapter, within the required driver voltage range of 8 to 35 V. Step and direction pins were linked to designated Arduino ports, and the Arduino itself was powered via USB.

To facilitate the pulsatile motion and control the output waveform, a cam/cam follower mechanism is created to couple the mechanical rotational motion of the stepper motor input to a mechanical translational motor, which compresses a custom bulb pump [Figs. 5(c)–5(e)]. The design of the cam profile directly determines the output profile of the resultant PPG curve. Coupled with an outlet pressurization mechanism, the output PPG profile is defined by three key features: amplitude (A) and period (T) of the primary and secondary peaks and the total waveform period. The total period is directly proportional to the rotational speed of the stepper motor shaft, which is programmable through the Arduino. The phase between the primary and secondary peaks is directly controlled by the separation of the peaks on the cam profile, where the cam profile is defined in degrees (0 deg to 360 deg) with one revolution completing one pulse period. The amplitude (A) is a function of surface profile, outlet pressurization, and the cam radius. The outlet pressurization is controlled by a roller mechanism that compresses the outlet following the phantom [flow gate in Fig. 5(f)] in three different stages to create different flow restrictions via tubing compression.

### 2.3 Blood-Mimicking Fluid

Blood-mimicking fluid (BMF) is created using a mixture of India ink and deionized water^27^ to capture the optical absorption of whole blood at 660 nm. Arterial blood typically has an oxygen saturation level ranging between 95% and 100%.^46^ The desired absorption coefficient for the BMF at this level of oxygenation ranges between 2.49 and $1.71\;{\mathrm{cm}}^{-1}$ for 95% and 100%, respectively. The BMF concentration is created using a stock solution of 40.84 g of deionized (DI) water and 0.0812 g of India ink to create an initial concentration of 0.198% by weight. Measurements for the optical absorption spectrum of the solution are performed on a Cole-Palmer VIS spectrophotometer.

## 3 Results

### 3.1 Wrist Phantom and BMF

Developing a silicone wrist phantom through 3D printing and silicone casting provides the advantage of the flexibility and uniformity of silicone-casted material, as well as the versatility of geometrical design and modification of additive manufacturing. Different combinations of silicone mixtures with varying ratios of solution A and solution B were performed and evaluated for mechanical elasticity. The results of mixture variations and elastic modulus are shown in Table 3.

**Table 3:** Mechanical characterization of casted silicone.

Cast ratio (B:A)	E (kPa)	Approximate cure time (h)
20:80	42.6±6.9	24+
30:70	100.3±7.3	16
40:60	151±21	12
50:50	240±9.2	6
60:40	193±15	5
70:30	22.3±5.3	2

Mechanical testing was performed on various silicone formulations to identify a material with an elastic modulus representative of the radial artery. Although several mixtures were evaluated, the final material selected for the vessel was a custom-cast silicone with a 50:50 ratio (manufacturer recommended), yielding an elastic modulus of ∼240  kPa. This formulation was chosen for its manufacturability, flexibility, and mechanical properties falling within the range for typical tissue elasticity.

The wrist phantoms are created for the three Fitzpatrick skin tone ranges (FST I to II, FST III to IV, and FST V to VI) and different obesity levels representing arterial depth (2.5 and 5.0 mm).

**Fig. 6:**
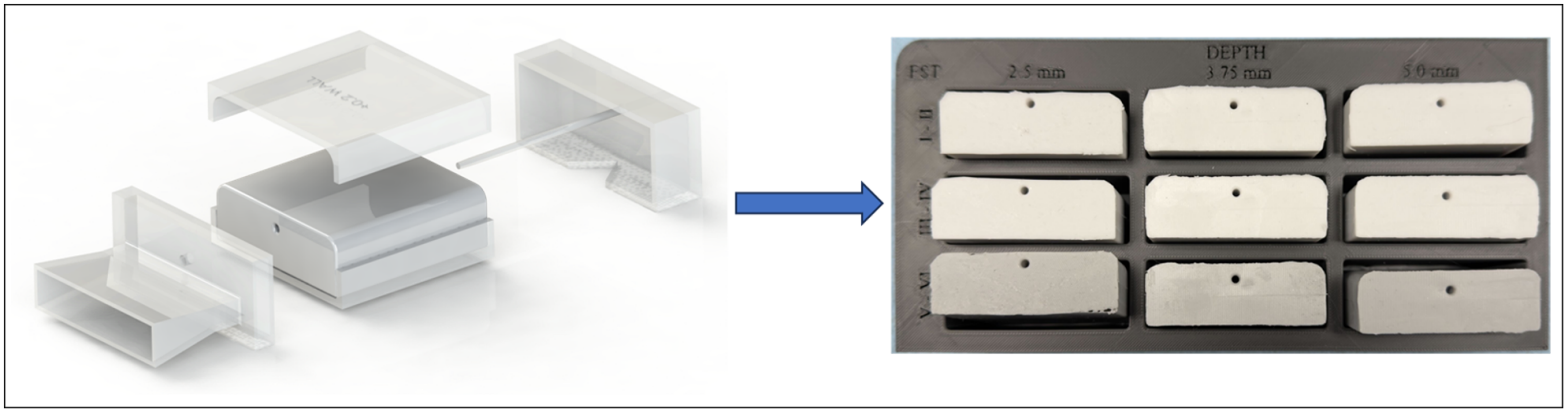
Phantom casting mold model to finished product. Wrist phantoms ranging from shallow to deep vessel location (increasing BMI, varying across each column, column labels 2.5, 3.75, 5.0 mm) and different Fitzpatrick skin tones (I to VI, varying down each row, row labels I to II, III to IV, and V to VI).

The mold casting process and results for the optical phantoms are shown in Fig. 6. The optical properties of the wrist phantoms were captured using an integrating sphere. Each phantom is created following a calibration set of 2.0 in. diameter by 2 mm thickness phantoms to compensate for interdependence between the absorber and scatterer used to achieve the desired optical properties. Eqs. (1) and (2) are used to estimate the amount of carbon black and titanium dioxide concentrations required to achieve the desired optical properties

$$\mu_{a}\;[{\mathrm{cm}}^{-1}]=3.2\times 10^{4}\;[C_{\mathrm{CB}}]-0.017,$$ (1)

$$\mu_{s}^{\prime }\;[{\mathrm{cm}}^{-1}]=5.5\times 10^{3}\;[C_{{\mathrm{TiO}}_{2}}]+2.3.$$ (2)

Optical properties measurement (Table 4) shows the absorption coefficient range for each thickness of the optical disc test phantom.

**Table 4:** Experimental optical properties for Fitzpatrick skin tone range at 660 nm of 51.2 mm by 2 mm thickness disc phantoms.

Fitzpatrick skin tone	Optical properties at 660 nm (${\mathrm{cm}}^{-1}$)		Mass (g)
	$\mu_{a}$	$\mu_{s}^{\prime }$	
I to II	0.39±0.02	16.7±0.9	3.411±0.002
III to VI	0.49±0.02	23.3±0.9	3.4113±0.0006
V to IV	1.8±0.2	22.4±2	3.413±0.002

**Fig. 7:**
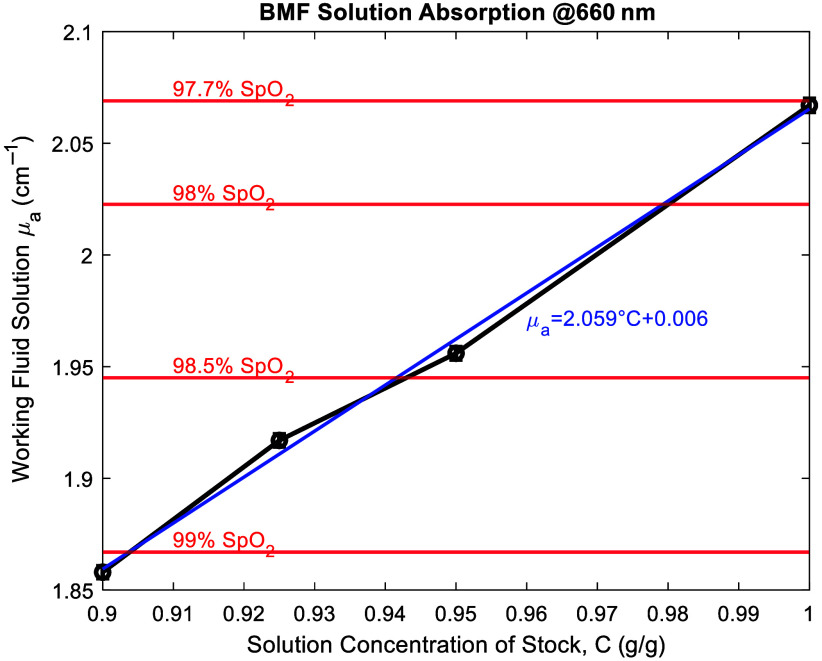
Concentration curve created for blood-mimicking fluid for a range of India ink and DI water mixtures for a desired optical absorption coefficient.

The BMF is developed using a combination of India ink and water to capture the whole blood absorption coefficient target at 660 nm wavelength. Figure 7 provides a concentration curve for the BMF and the absorption spectrum of the fluid used in the pulsatile phantom system.

The calibration curve equation for the BMF is used to target a blood oxygen saturation range of ∼97% to 99%, which is equal to an absorption coefficient of 2.18 to $1.87\;{\mathrm{cm}}^{-1}$, respectively. The concentration used for the resultant BMF is the combination of 465 g of DI water and 0.382 g of Higgins Waterproof Black India Ink (Fl Oz 29.6 mL/cc No. 44201). The resultant BMF is created and measured using a spectrophotometer (Fig. 7), and at 660 nm, the absorption coefficient is measured to be $2.071\pm 0.003\;{\mathrm{cm}}^{-1}$. This is equivalent to the absorption coefficient of whole blood at 97.7% oxygen saturation.

### 3.2 Pulsatile Flow System and PPG Measurement

**Fig. 8:**
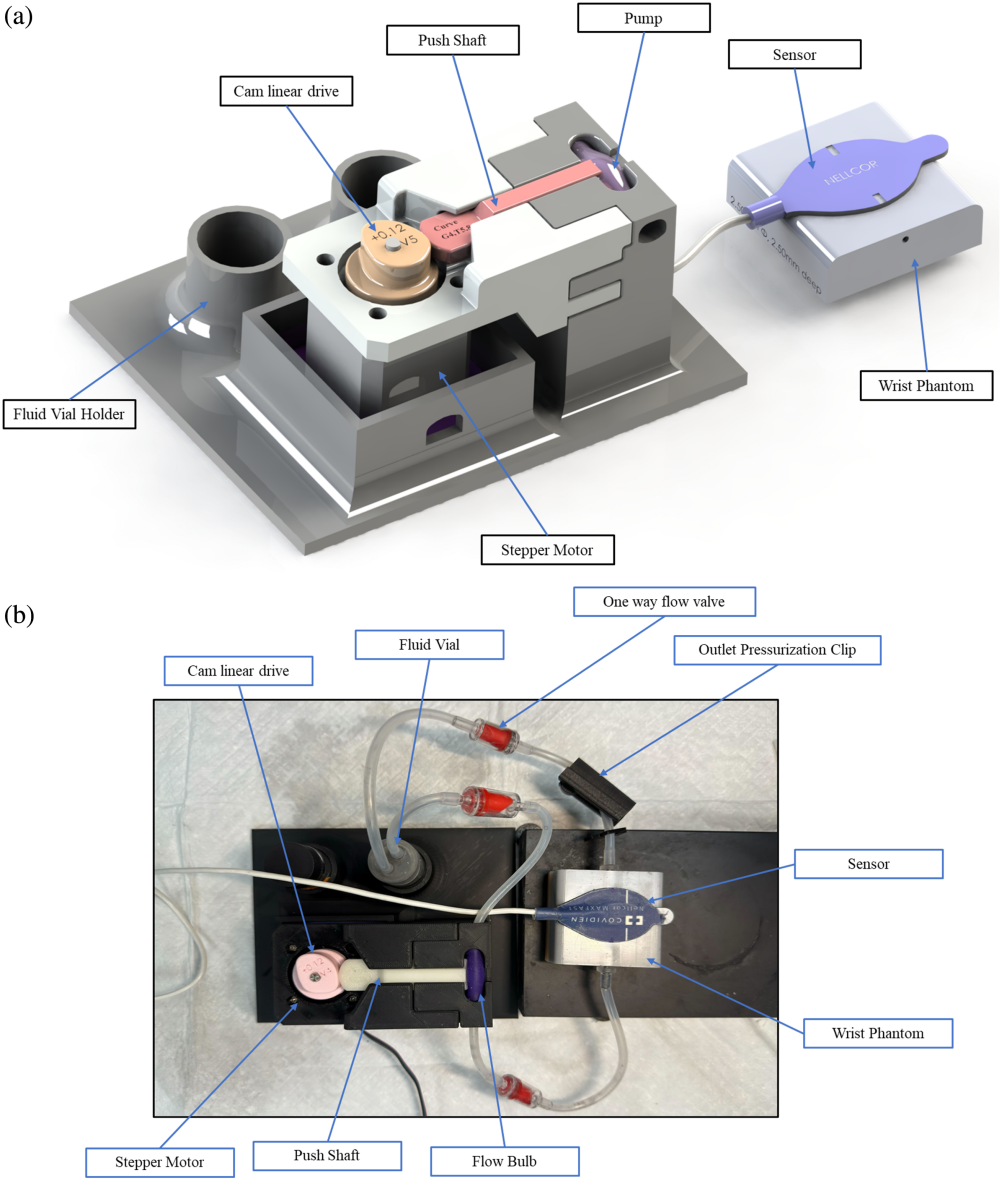
(a) Render model of the phantom system with labeled components and (b) top view image of the phantom with labeled components.

The resultant dynamic phantom system is shown in Fig. 8. The phantom comprises a base plate with two BMF vial holders, a motor mount, and a pump mechanism tower. The motor is placed in its housing and secured with a top mounting piece by four screws. The pump bulb is fitted with silicone tubing into the top slot, the push shaft is slipped in, and the cam is secured onto the motor shaft. In Fig. 8(b), two additional components are observed that are not represented in the model: three one-way flow valves and the outlet pressurization clip.

**Fig. 9:**
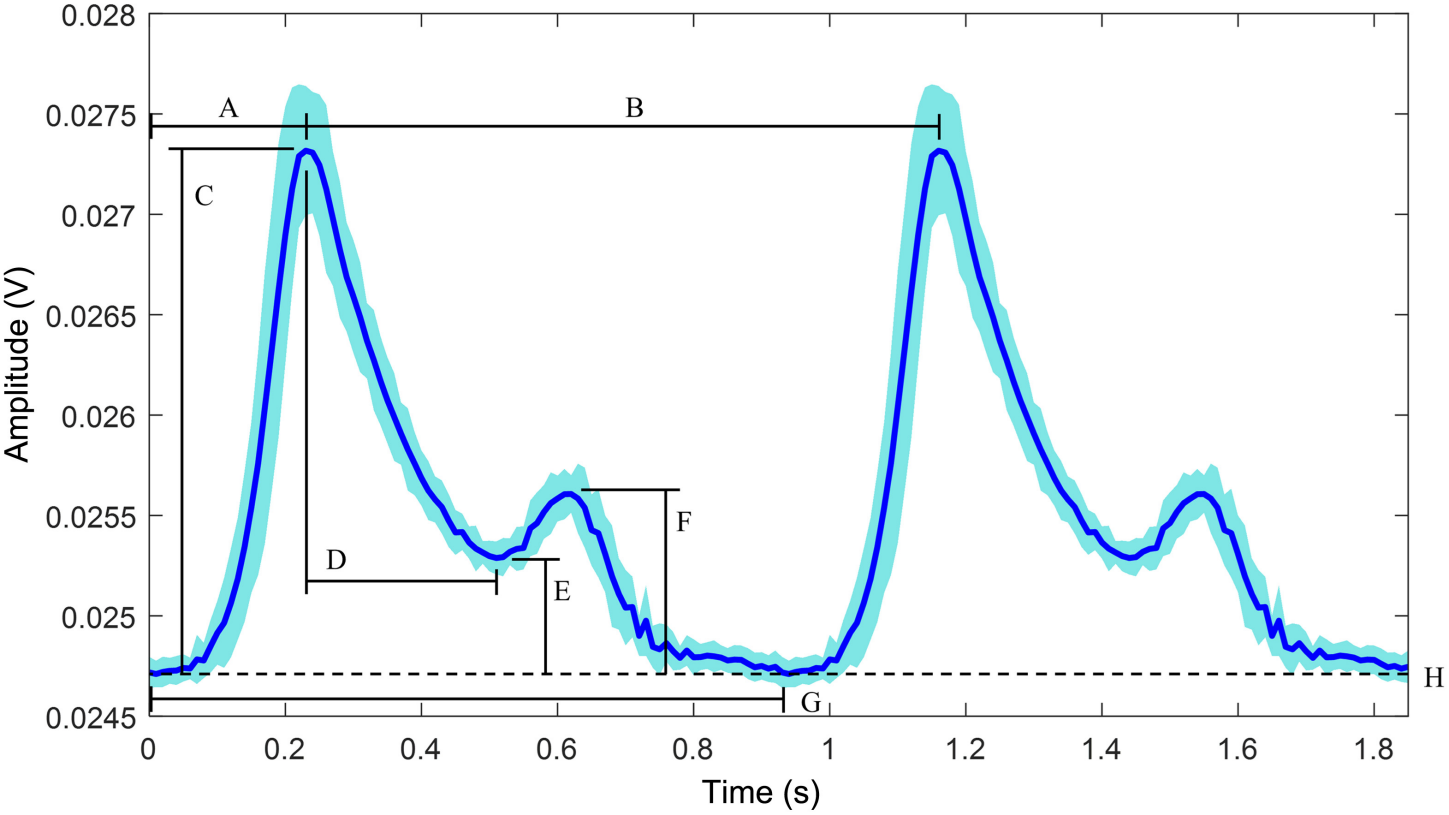
Experimental waveform (blue line) with variation band of 1 standard deviation (light blue shade). The waveform is captured from the baseline condition (lightest skin tone and shallowest vessel depth).

The phantom system can achieve pulse rates ranging from 45 to 174 beats per minute (BPM), controlled through an Arduino. The system is powered through a 12 V DC power supply to the stepper motor, and the Nellcor PPG sensor is connected to an AFE44x0SPO2EVM evaluation board from Texas Instruments (TI, Dallas, Texas, United States). The measured waveform is shown in Fig. 9 with a mean waveform and deviation band alongside labeled PPG features (Table 5). Table 5 provides quantitative values for key morphological features of the PPG waveform, such as systolic amplitude, diastolic amplitude, and timing intervals, which are commonly used in physiological assessments and algorithm development for wearable health monitoring.^47^ These features are relevant for estimating cardiovascular parameters such as heart rate variability, vascular compliance, and blood pressure.

**Table 5:** PPG waveform feature labels for Fig. 9.

Label	Value	Description
A	0.23 (s)	Systolic incline phase
B	0.93 (s)	Peak-to-peak cardiac period
C	0.0026 (V)	Systolic amplitude
D	0.28 (s)	Systolic decline
E	0.0005 (V)	Dicrotic notch height
F	0.0009 (V)	Diastolic amplitude
G	0.93 (s)	Waveform period
H	0.0247 (V)	DC baseline value

The pulsatility index of the measured baseline PPG waveform is 10.2%. Baseline is taken to be the phantom with the lightest skin tone and shallowest vessel depth. This is comparable to literature values that have reported human pulsatility index ranging from 0.2% to 20%.^11^^,^^48^ This can be calculated using the equation as follows:

$$\mathrm{PI}\%=\frac{\max-\min}{\text{mean}}\times 100\%.$$ (3)

The custom pumping action of the bulb creates a compression of $1050\;{\mathrm{mm}}^{3}$ volumetric area. During a full compression phase while running the stepper motor at 1 RPM, this generates an approximate peak volumetric flow rate of $5250\;{\mathrm{mm}}^{3}\;s^{-1}$ given a systolic incline phase of 0.2 s. A pressure transducer is fitted initially to assess the flow in the phantom system; the pressure is observed to be in the physiological range in the phantom inlet, ranging between 160 and 40 mmHg. At the outlet, after the pressurization clip, the pressure is observed to decrease and remain between 10 and 20 mmHg. The pressure is measured using a Living Systems PM-4 Perfusion monitor. The PPG waveform profile is manipulated by combining the cam radius and profile and outlet pressurization stage.

**Fig. 10:**
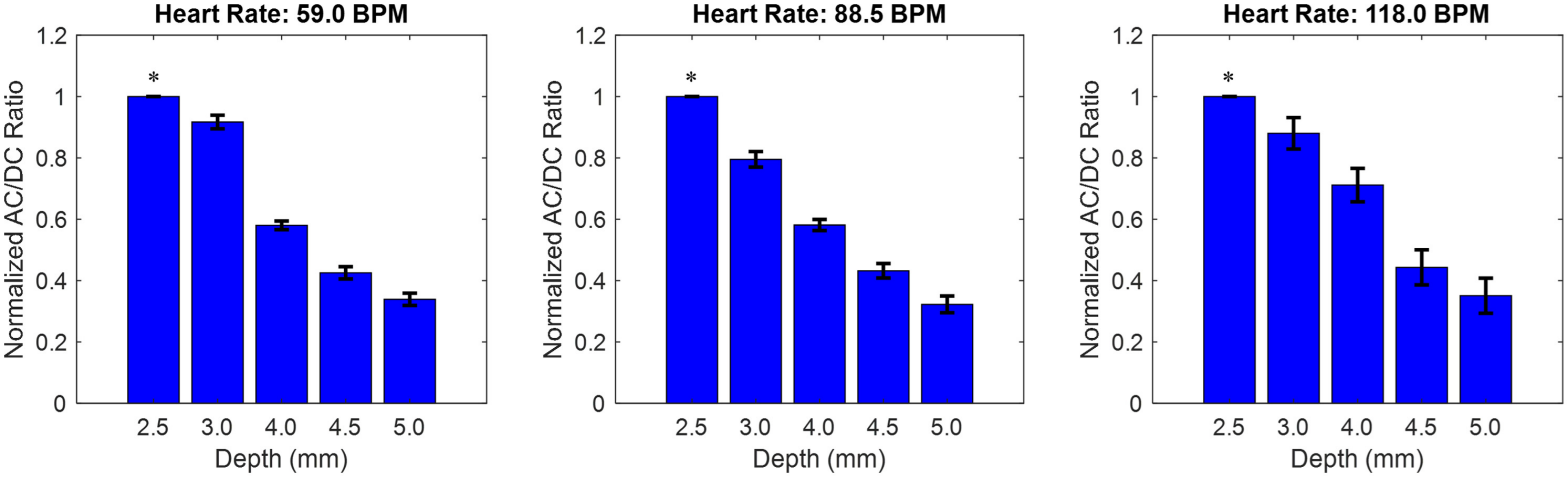
AC/DC ratio comparison across depths for each heart rate measured, at three BPM on Fitzpatrick skin tone V to VI phantoms. *Baseline is 2.5 mm arterial depth.

Various wrist phantoms are created to evaluate the effects of skin tone and obesity on the PPG signal. Skin tone is altered by changing the optical properties, and the obesity level is captured by changing the vessel depth, ranging from 2.5 to 5 mm. A comparison of the AC/DC ratio is shown in Fig. 10.

Figure 10 shows the change in the AC/DC ratio as vessel depth increases. The trend is consistent across all three heart rate frequencies. For each heart rate, the change in AC/DC ratio from baseline is compared, showing up to a decrease of 67.7% in the overall AC/DC ratio at the deepest vessel. A comparison of these AC/DC ratio percentages is shown in Table 6.

**Table 6:** Comparison of AC/DC ratio percentage changes using the baseline of 2.5 mm vessel depth at each depth.

Heart rate (BPM)	Depth (mm)				
	2.5 (Baseline)*	3.0	4.0	4.5	5.0
59	0	8.31	42.0	57.5	66.1
88.5	0	20.5	41.9	56.8	67.7
118	0	12.0	28.9	55.7	64.9

* Values are percentage (%) change from baseline.

Changes to the depth at each frequency demonstrate a range from 8% to 20% change at the first vessel depth of 3.0 mm and a maximum change of 64% to 67% at the lowest vessel depth of 5.0 mm.

**Fig. 11:**
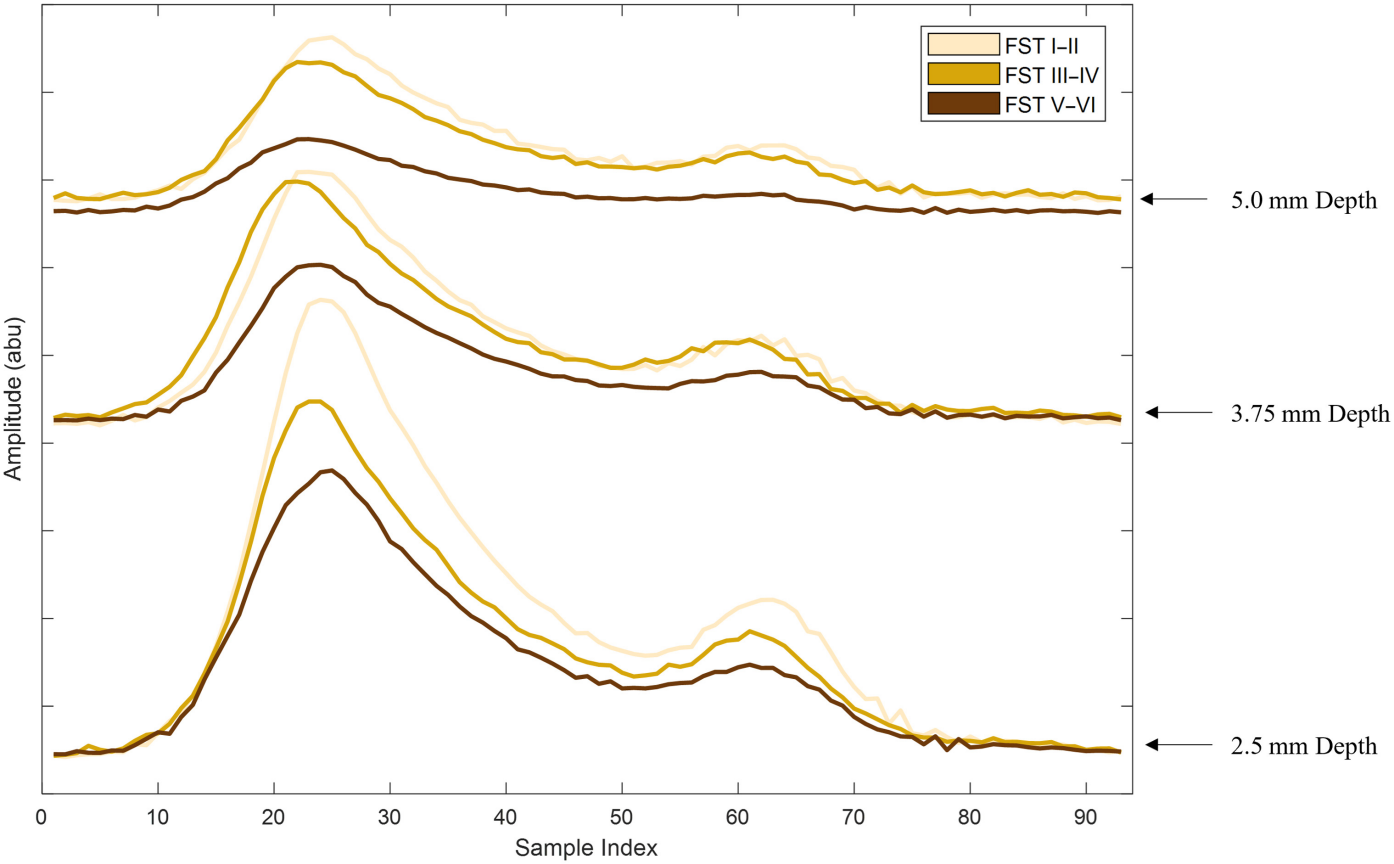
Example waveforms for 3 depths for each Fitzpatrick skin tone range from I to II, III to IV, and V to VI. Each waveform is normalized and grouped by depth to each respective baseline (the strongest signal).

Figure 11 provides an example of the nine waveforms captured for each of the phantoms representing the various vessel depth and skin tone ranges. Each waveform has been grouped by depth.

**Fig. 12:**
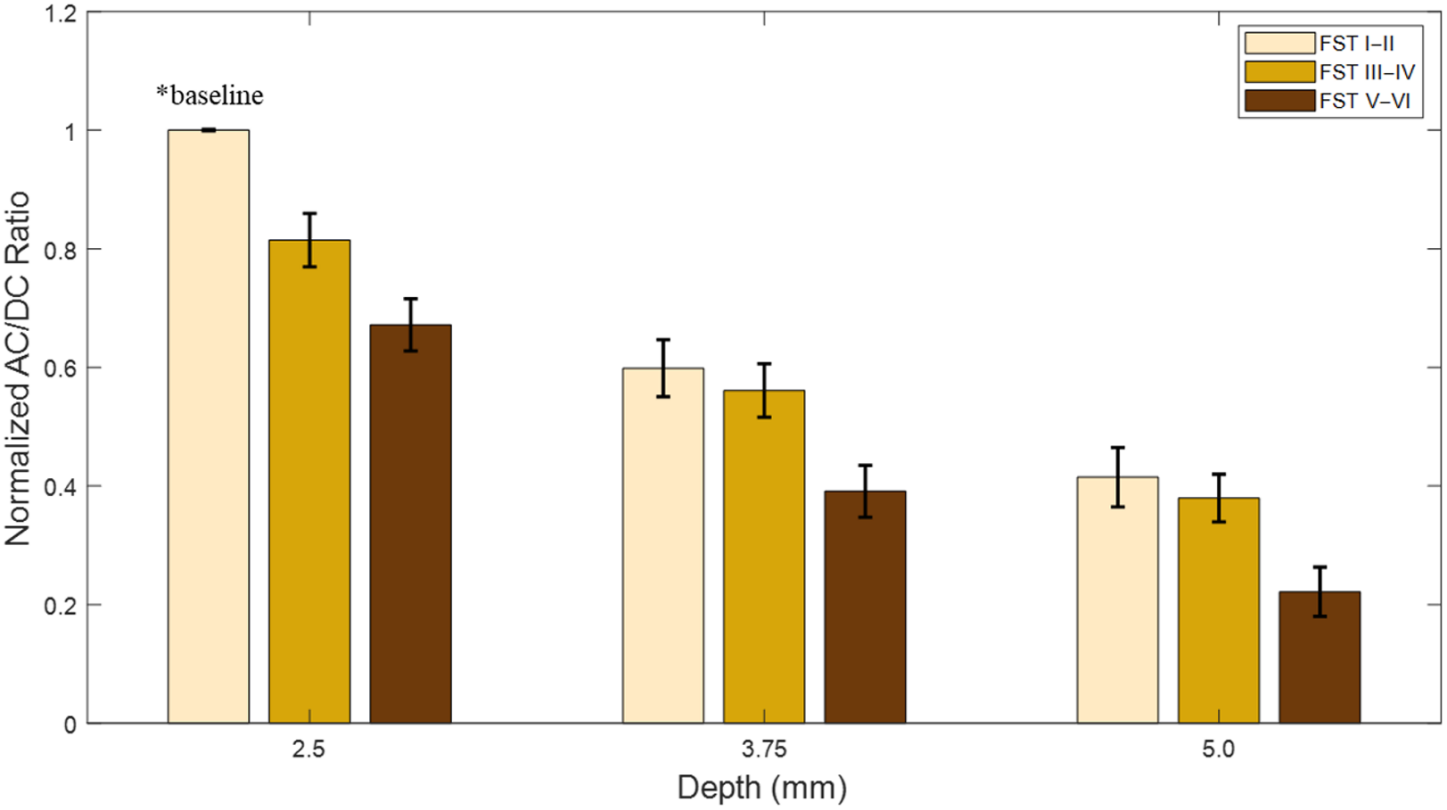
Normalized AC/DC ratio comparison of 3 depths for each Fitzpatrick Skin Tone range from I to II, III to IV, and V to VI. *Baseline value is skin tone I to II and 2.5 mm arterial depth.

Figure 12 illustrates data captured from nine different phantoms (Fig. 6), ranging from skin tone I to II with the shallowest arterial depth of 2.5 mm, an intermediate depth of 3.75 mm, and the deepest artery at 5.0 mm, to the darkest skin tone V to VI. Here, at each artery depth, as expected, the AC/DC ratio of the pulsatile waveform decreases from the lightest skin tone to the darkest. It is observed that for the 3.75 and 5.0 mm vessel depth, there is a larger decrease among all skin tones (I to II and III to VI) versus the darkest skin tone (V to VI) than there is among skin tones I to II and III to IV. The AC/DC ratio value at the lightest skin tone (I to II) shows values that fall within the range of the AC/DC ratio of the darkest skin tone (V to VI) if the previous, shallower depth. Relative to the baseline taken for skin tones I to II and 2.5 mm vessel depth, the maximum AC/DC ratio decrease of the darkest skin tone (V to VI) and 5.0 mm is 77.8%. Table 7 highlights the changes to the AC/DC ratio.

**Table 7:** AC/DC ratio change between each phantom and baseline (2.5 mm depth, I to II).

Fitzpatrick skin tone	Depth (mm)		
	2.5	3.75	5.0
I to II	0 (baseline)*	40.1	58.5
III to VI	18.5	43.9	62.0
V to IV	32.8	60.9	77.8

* Values are percentage (%) change from baseline.

A qualitative test using an Apple Watch Ultra 2 was performed to assess whether a commercial wearable device could detect a PPG signal from the phantom without any hardware or algorithm modifications. The watch was placed directly on the phantom surface without securing the straps/bands. Although not designed to analyze or extract raw data from the device, this test served to verify whether the signal strength and heart rate generated by the phantom were sufficient for detection by an unmodified consumer device. Heart rate readings displayed by the watch were recorded and found to correspond to the programmed driving frequencies (59, 89, and 118 BPM), indicating that the phantom output falls within the detectable range of commercial wearables.

## 4 Discussion

The PPG signal strength decreases with increasing vessel depth and with increasing optical properties. The most critical observation arises from the comparison of the AC/DC ratio between skin tones and across vessel depth. The AC/DC values between the lightest skin tones (I to II) of a deeper vessel and the values of the darker skin tones (V to VI) of the previous vessel depth fall within the same range. This effect appears most significantly between skin tones V to VI, depth 3.75 mm, and skin tones I to II, depth 5.0 mm. This could lead to errors associated with the interactions between obesity (vessel depth) and skin tone. The AC/DC ratio is used as a general metric to compare overall waveform quality. In this study, we used it to evaluate how changes in optical absorption, scattering, and vessel depth influence PPG signals while holding mechanical behavior constant under controlled and repeatable conditions. As wearable health technologies increasingly rely on PPG-derived features to estimate parameters such as blood pressure, vascular stiffness, and oxygen saturation, accurate waveform feature extraction becomes critical. Many of these algorithms, particularly those involving machine learning, depend on consistent detection of waveform amplitudes and timing features. When both skin tone and vessel depth increase, the compounded reduction in AC/DC ratio can obscure these features, potentially leading to misclassification or reduced accuracy in commercial systems. Our phantom system allows for the controlled investigation of such interactions, which are difficult to isolate in human populations and can support future algorithmic development to mitigate bias and improve performance. Although the current study does not include direct human subject data, previous computational modeling work by our group has demonstrated that increases in skin pigmentation and obesity (modeled as vessel depth) reduce AC/DC ratio and signal quality,^8^^,^^9^ which aligns with the trends observed in the phantom system. The primary aim of this work is to develop a modular and tunable phantom that can simulate these physiological variations and serve as a ground truth testing platform for future validation against empirical human data. Importantly, these reductions in AC/DC ratio have been implicated in reduced accuracy of commercial wearable devices as diminished pulsatile signal strength can affect algorithms for biomarker estimation.

Figure 2 presents an overview of the full design considerations explored during the conceptual phase of phantom development. Although not all parameters, such as layered optical properties and skin surface curvature, were implemented in the final prototype, they remain part of our ongoing efforts to improve physiological realism in future iterations. These features were included in the figure to communicate the broader design framework and potential for future expansion.

Although Fig. 9 presents the waveform at the baseline frequency, the general waveform shape remained consistent at higher driving frequencies of 88.5 and 118 BPM. However, the standard deviation within each waveform was larger at higher BPMs, likely due to transient effects in the flow system. Increased pump speed can introduce nonuniformities in flow delivery before all system components reach steady-state operation, with cam follower dynamics, tubing elasticity, and fluid inertia all contributing to reduced stability. The AC/DC ratio variation for these higher BPMs, relative to their respective baselines, is shown in Fig. 10. Here, larger variations are observed with increasing speed, which may result from the combined influence of greater transient effects at higher speeds and reduced waveform signal quality at greater vessel depths. As vessel depth increases, a smaller fraction of photons contributing to the dynamic signal reaches the detector, potentially amplifying variability in the AC/DC ratio. Given the geometry of the sensor (NellCor) and the given optical properties, Table 6 suggests that there is a greater variability in the interaction of the dynamic signal at shallower depths compared with deeper vessels. The sensor has a source–detector separation of ∼10  mm. This could indicate higher sensitivity to minor changes in system configuration, such as sensor alignment or other environmental factors. This could suggest that intermediate depths may represent a transition zone where the interplay between optical attenuation and signal detectability is most sensitive to experimental variability. This can help provide guidelines for device design modifications to adjust properties according to system performance across desired depth and skin types.

With respect to the heterogeneity of the tissue, the skin is commonly modeled with various layers; however, with respect to the detector, this component simply captures the intensity of incoming light. As a result, we have used a bulk set of optical properties^45^ to create our wrist phantom. These bulk properties are derived from experimental models, including both *in vivo* and *ex vivo* measurements of the skin. At the wavelength of interest, an increase in vessel depth due to the fat layer is a better representation of obesity-induced changes, given that hemoglobin is the predominant absorber in the dynamic signal. Regarding smaller superficial vasculatures, these vessels vary significantly in size, shape, and distribution across individuals and typically range from tens to hundreds of micrometers in diameter. Although they contribute to the overall optical scattering and absorption of tissue, our study focuses on modeling the primary PPG signal generated by the pulsatile radial artery.

As for changes in optical properties due to varying levels of obesity, these remain incompletely characterized in the literature. Obesity can lead to a range of physiological changes, including increased subcutaneous fat, altered vascular density, and changes in skin hydration and thickness as we have introduced in our previous work.^7^ However, due to the lack of standardized, quantitative optical property data specifically tied to obesity-related adipose tissue changes, we modeled obesity through an increase in arterial depth, an effect that has been supported by both anatomical and computational studies. Future iterations of the phantom can incorporate layer-specific optical properties and heterogeneous tissue structures as more data become available. The current design and modeling approach provides a tractable and reproducible system for assessing first-order effects on PPG signal strength under controlled conditions.

When driving the motor at each frequency, a specific time delay is set in the programming of the motor; in this case, the motor is programmed to a rotational frequency of 1.0, 1.5, and 2.0 Hz, corresponding to 60, 90, and 120 BPM. We have used these three frequencies to drive the motor as our primary analysis frequencies given typical heart rates at rest without strenuous activity. An analysis frequency must be specified to divide the overall signal to separate individual waves when analyzing the captured PPG waveform. It is seen that the analysis frequency and the captured heart rate are slightly lower than the set theoretical values given to the motor. The heart rate for each analysis frequency is 59, 88.5, and 118 BPM. The motor shaft can be driven at rotational speeds up to 240 RPM; however, when the pump system is fully assembled, the maximum heart rate reaches ∼174 BPM. This is most likely a result of the friction of various mechanical components in the system and any losses in power transfer between the different domains and components; these losses seem to increase with frequency.

The critical factor in the mechanical performance of biological tissue is the viscoelastic nature of the mechanical behavior under applied strain, particularly the nonlinear and time-dependent mechanical response of arterial walls under physiological loading. This is a major limitation of many tissue phantoms, including the one presented in this paper. The path across the stress–strain relationship of the large arteries is primarily determined by the interactions between collagen and elastin content and their behavior under the physiological pressure ranges. The forward path shows behavior more characteristic of elastin than past a certain strain region; higher stiffness behavior characteristics of collagen can be seen. During the relaxation phase, the tissue exhibits a certain viscous behavior. Our phantom currently models elastic compliance but does not incorporate viscous damping characteristics. As a result, certain dynamic features of the PPG waveform, such as delayed recoil or waveform broadening due to hysteresis, may not be fully replicated. However, the primary goal of this study was to isolate optical effects (e.g., skin tone and vessel depth) under controlled mechanical conditions. Future iterations may incorporate viscoelastic vessel materials or damping layers to more accurately reflect tissue biomechanics.

Although arterial and surrounding tissue stiffness can vary with factors such as age and pathology, the scope of this work was to first establish a baseline phantom system with consistent mechanical properties to isolate the effects of optical properties and vessel depth on PPG signal behavior. During development, a range of silicone and 3D-printed materials were tested for their mechanical properties to inform phantom fabrication. Stiffer materials, such as those produced through 3D printing, were characterized but not implemented in this initial study. However, to develop phantoms of different stiffness, this can be achieved using the methods outlined. The modular design of the phantom supports future integration of variable mechanical properties, enabling expanded investigation into the effects of tissue compliance on PPG signal quality.

The interaction of the cam and the outlet pressurization must be considered to create the desired wave shape at the PPG output. The outlet pressurization is biologically representative of the pressure drop and increased fluid resistance as arteries transition into arterioles and capillaries. As the arteries undergo this transition and become smaller and smaller in diameter, the pressure inside decreases to maintain the structural integrity of the vessels. It is also observed that the pulsatility of the flow is lost in the post-pressurization outlet, with pressure falling to ∼10 to 20 mmHg. This range is physiologically representative of the pressure seen inside the venous system.^49^ Although pressure is initially monitored to ensure the system remains within the physiological range, the pressure is not measured or controlled during all other processes. The addition of a pressure measurement node requires the addition of other components that create changes in the flow area due to the fitting size and components of the PM-4 monitor. Controlling the pressure in the system, as it currently stands, relies on the design of the cam profile, drive frequency, and the size of the bulb pump. Physiological blood flow in the radial artery was measured with a Doppler ultrasound and is observed to range between 1000 and $7000\;{\mathrm{mm}}^{3}\;s^{-1}$ depending on the individual. The system provides flow within this range; however, the limitation exists that multiple system components would need to be altered to achieve the same flow rate but control the total pressure inside the wrist phantom. A mechanism could be implemented at the inlet of the wrist phantom to control the pressure of the fluid as it enters the wrist phantom, considering the change in the diameter of the tubing in the system and the phantom’s radial artery diameter. These are all further considerations that can be made as this system undergoes further design iterations.

Contact pressure plays a critical role when taking data with all the devices tested on the phantom. With the Nellcor, a mass of 60 g with a square surface area of $60\times 60\;{\mathrm{cm}}^{2}$ was placed on top of the Nellcor sensor during data acquisition. This provided a consistent contact pressure that a sensor at the wrist would experience. This ensures that sufficient and constant contact pressure is maintained throughout the acquisition process. It should be noted that the Apple Watch Ultra 2 (natural titanium) has a similar mass of 61.4 g as reported on Apple’s website. When testing with the Apple Watch, the wristbands were not used to secure the watch to the phantom; this created some difficulties with trying to achieve proper contact between the watch sensors and the phantom as the underside of the Apple Watch has a prominent curvature for the glass. Although the Apple Watch is intended for superficial PPG with its green LEDs, it does contain LEDs in the longer near-infrared (IR) range. It was observed that the Apple Watch could report the correct heart rate, matching the driving frequency of the motor, for the darkest (type V to VI) phantoms. These were qualitative observations made during testing; further exploration of PPG signals captured by commercial systems, which allow access to raw data, would greatly benefit the community.

Alongside contact pressure, many environmental factors were observed to affect the PPG signal from the Nellcor. To minimize these effects, all data on each plot were captured in a dark room and all in the same session. Changes to environmental illumination, such as room lights, other monitor screens, or LEDs on evaluation boards/Arduinos, can all cause interference with the Nellcor signal. Other mechanical noise, such as vibration from the motor or motions on the test bench, could also cause shifts in the data. Therefore, it is critical to isolate all these sources of error.

The current system is tuned to 660 nm wavelength (one of the wavelengths of the NellCor commercial ${\mathrm{SpO}}_{2}$ sensor). Our goal is currently to observe and evaluate the effects of changes to optical properties and vessel depth on the PPG waveform, not specifically ${\mathrm{SpO}}_{2}$. As for the current design, both the working fluid and the silicone phantom are not developed for other wavelengths. Equations have been developed that allow for tuning the phantom and fluid to match the optical properties at other wavelengths. Multiwavelength capabilities are limited to the properties of the base material, as well as carbon black and titanium dioxide (the absorber and scatterer agents).

## 5 Conclusion

This work successfully demonstrates the development of a dynamic, customizable pulsatile phantom designed to simulate the radial artery’s PPG waveform at the wrist. By replicating human tissue’s mechanical, optical, and geometrical properties, the phantom serves as an invaluable tool for testing and refining wearable health devices. By integrating a cam/cam follower mechanism, a custom bulb pump, and tailored wrist phantoms, this design accommodates skin tone and vessel depth variations to emulate realistic conditions influenced by factors such as obesity and skin pigmentation, elements that profoundly impact PPG signal quality.

This phantom addresses a critical gap in current wearable health technologies, which often struggle to provide accurate readings across diverse demographic groups. By mimicking the effects of tissue composition and physiological variations on PPG signals, the phantom enables a more comprehensive evaluation of device performance, particularly under conditions where current devices have shown discrepancies. As wearable technologies continue to advance, the demand for equitable and accurate health monitoring solutions grows. This dynamic phantom provides a robust platform for testing these devices in controlled, representative environments, ultimately contributing to more inclusive and accurate health assessments. By facilitating improvements in wearable technology across a broader spectrum of users, this work supports ongoing efforts toward health equity and the development of personalized healthcare solutions that are accessible and reliable for all populations.

## Data Availability

The code, component list/details, and data generated from this study can be made available from the corresponding author upon reasonable requests. The drawings and detailed part list of all major components for the pulsatile pump system are under submission to the US Patent Office (USPTO application # 19/222,638).
